# Analysis of Quantum Correlations Obtained Using Local Optimal Universal Asymmetric Cloners

**DOI:** 10.3390/e25010029

**Published:** 2022-12-23

**Authors:** Cătălina Cîrneci, Iulia Ghiu

**Affiliations:** 1 Faculty of Physics, University of Bucharest, 405 Str. Atomistilor, 077125 Magurele, Romania; 2 Research Institute of the University of Bucharest (ICUB), 90-92 Sos. Panduri, 5th District, 050657 Bucharest, Romania

**Keywords:** concurrence, quantum discord, asymmetric cloning

## Abstract

We apply the local optimal universal asymmetric cloning machine on an initially pure entangled state of two qubits. As output, we obtain two final states which present quantum correlations. We analyze three types of quantum correlations among the final states, namely, concurrence, quantum discord, and consonance. A detailed comparison between concurrence, quantum discord, and consonance is made, and we find that consonance is greater than quantum discord, which is in turn greater than concurrence.

## 1. Introduction

Quantum technologies have become a widely used expression in the last few years. They involve a large number of branches of physics: quantum information, quantum computing, quantum optics, quantum communication, etc. All these areas are based on an important number of resources, such as: entanglement [1,2,3,4,5,6,7,8,9,10,11,12,13,14,15,16,17,18,19,20,21,22,23,24,25], nonclassicality [26,27,28,29,30,31], non-Gaussianity [32,33,34,35], quantum discord [36,37,38,39,40,41,42], and quantum coherence [43,44,45,46,47,48,49].

Our purpose in this paper is to investigate the behaviour of three kinds of quantum correlations, namely, entanglement, quantum discord, and consonance, of the output states obtained from the protocol called asymmetric broadcasting of entanglement. Suppose that two spatially separated observers, Alice and Bob, share a two-qubit system found in the entangled state α|00〉+β|11〉. Each of them applies the local 1→2 optimal universal symmetric cloning machine [50] on this state, and they obtain two identical final states. The symmetric broadcasting of entanglement is realized when the output states are inseparable [51].

On the other hand, a different cloning machine was proposed, namely, one that generates two different clones but still keeps the information carried by the initial state. Such a cloning machine is known in the scientific literature as the 1→2 optimal universal asymmetric cloner [52,53]. The asymmetric cloning machine is important in the context of quantum key distribution in quantum cryptography. Namely, it was proven that in the case of asymmetric cloning, the best eavesdroping strategy is obtained [54,55,56]. With the help of this cloner, we have proposed the asymmetric broadcasting of entanglement [57,58] in the case when the initial state is a pure one. This concept was generalized to the case when the initial state is any inseparable two-qubit mixed state [59]. For the analysis of the broadcasting of entanglement in the previous articles, the Peres–Horodecki criterion [60,61] was employed, and no further investigations regarding the degree of entanglement were conducted.

The paper is organized as follows: In Section 2, we show that the two output states obtained in the protocol of asymmetric broadcasting of entanglement are *X* states and determine their concurrence. Section 3 is dedicated to the evaluation of the quantum discord of the states generated by the broadcasting of entanglement. We plot the quantum *A*-discord of these states in terms of the parameter α, which characterizes the initial state, and *p*, which describes the asymmetric cloner. The investigation of consonance is presented in Section 4, where we also illustrate the behaviour of this measure in terms of α and *p*. Furthermore, a detailed comparison between the concurrence, quantum *A*-discord, and consonance is presented. Our conclusions are outlined in Section 5. In Appendix A, we describe the approach of Li et al. [62] for computing the quantum discord for arbitrary *X* states.

## 2. The Concurrence of the States Obtained by the Local Optimal Universal Asymmetric Cloning Machines

**Figure 1:**
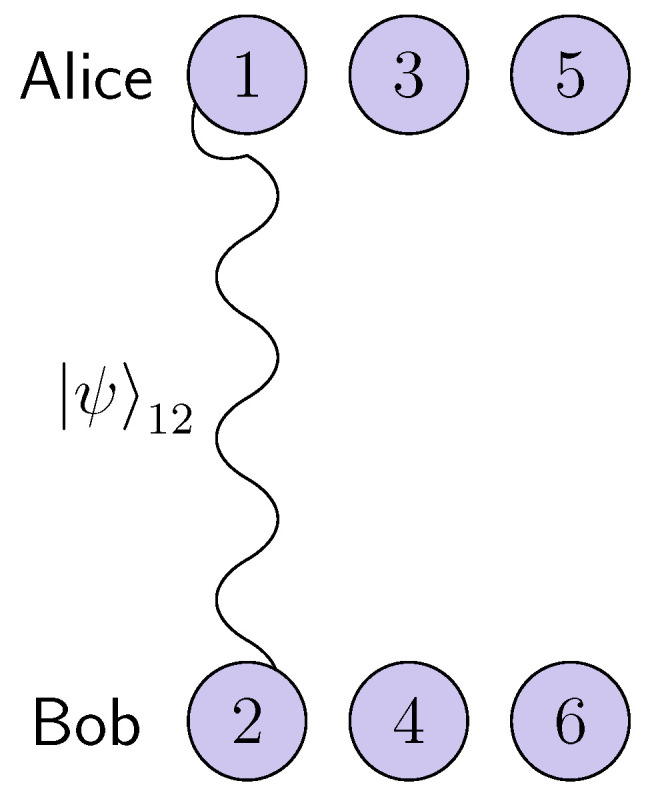
The initial state of the total system.

Consider that two spatially separated observers, Alice and Bob, share a two-qubit system found in the entangled state:

(1) $${|\;\psi \;\rangle }^{\left(12\right)}=\alpha |\;00\;\rangle +\beta |\;11\;\rangle ,$$

with α and β being complex such that ${\left|\alpha \right|}^{2}+{\left|\beta \right|}^{2}=1$. Alice also has two qubits found in the states ${|\;0\;\rangle }^{\left(3\right)}$ and ${|\;0\;\rangle }^{\left(5\right)}$, and Bob has two other qubits described by the states ${|\;0\;\rangle }^{\left(4\right)}$ and ${|\;0\;\rangle }^{\left(6\right)}$, as one can see in Figure 1.

Alice and Bob want to generate two inseparable states by locally applying the optimal universal asymmetric cloner on their qubits. This cloner is characterized by the unitary operator found in Ref. [57]:

(2) $$\begin{array}{c} U\left(p\right)|\;0\;\rangle |\;00\;\rangle =\frac{1}{\sqrt{1+p^{2}+q^{2}}}(|\;000\;\rangle +p|\;011\;\rangle +q|\;101\;\rangle ),\\ U\left(p\right)|\;1\;\rangle |\;00\;\rangle =\frac{1}{\sqrt{1+p^{2}+q^{2}}}(|\;111\;\rangle +p|\;100\;\rangle +q|\;010\;\rangle ), \end{array}$$

where p+q=1. N.B.: The symmetric cloner is obtained for p=1/2.

Let us denote by |ξ〉 the state obtained when the two observers apply the cloner U(p) characterized by an identical parameter *p* for both Alice and Bob:

(3) $$|\;\xi \;\rangle =U\left(p\right)⊗{U\left(p\right)|\;\psi \;\rangle }^{\left(12\right)}{|\;00\;\rangle }^{\left(35\right)}{|\;00\;\rangle }^{\left(46\right)}.$$

The two states $\rho^{\left(14\right)}$ and $\rho^{\left(23\right)}$ shared by Alice and Bob are given by the reduced density operators:

(4) $$\begin{array}{ccc} \rho^{\left(14\right)} & = & {\mathrm{Tr}}_{2356}\left|\;\xi \;\rangle \langle \;\xi \;\right|=\frac{1}{{\left(1+p^{2}+q^{2}\right)}^{2}}\{[p^{2}q^{2}+{\left|\alpha \right|}^{2}\left(1+p^{2}+q^{2}\right)]|\;00\;\rangle \langle \;00\;|\\ & & [p^{2}q^{2}+{\left|\beta \right|}^{2}\left(1+p^{2}+q^{2}\right)]|\;11\;\rangle \langle \;11\;|+4pq\alpha \beta^{*}\left|\;00\;\rangle \langle \;11\;\right|+4pq\alpha^{*}\beta |\;11\;\rangle \langle \;00\;|\\ & & +{\left(|\beta \right|}^{2}q^{4}+{\left|\beta \right|}^{2}q^{2}+{\left|\alpha \right|}^{2}p^{4}+{\left|\alpha \right|}^{2}p^{2})|\;01\;\rangle \langle \;01\;|\\ & & +(|\beta {|}^{2}p^{4}+|\beta {|}^{2}p^{2}+|\alpha {|}^{2}q^{4}+|\alpha {|}^{2}q^{2})|\;10\;\rangle \langle \;10\;|\} \end{array}$$

and

(5) $$\begin{array}{ccc} \rho^{\left(23\right)} & = & {\mathrm{Tr}}_{1456}\left|\;\xi \;\rangle \langle \;\xi \;\right|=\frac{1}{{\left(1+p^{2}+q^{2}\right)}^{2}}\{[p^{2}q^{2}+{\left|\alpha \right|}^{2}\left(1+p^{2}+q^{2}\right)]|\;00\;\rangle \langle \;00\;|\\ & & [p^{2}q^{2}+{\left|\beta \right|}^{2}\left(1+p^{2}+q^{2}\right)]|\;11\;\rangle \langle \;11\;|+4pq\alpha \beta^{*}\left|\;00\;\rangle \langle \;11\;\right|+4pq\alpha^{*}\beta |\;11\;\rangle \langle \;00\;|\\ & & +{\left(|\beta \right|}^{2}p^{4}+{\left|\beta \right|}^{2}p^{2}+{\left|\alpha \right|}^{2}q^{4}+{\left|\alpha \right|}^{2}q^{2})|\;01\;\rangle \langle \;01\;|\\ & & +(|\beta {|}^{2}q^{4}+|\beta {|}^{2}q^{2}+|\alpha {|}^{2}p^{4}+|\alpha {|}^{2}p^{2})|\;10\;\rangle \langle \;10\;|\}. \end{array}$$

A density operator is said to be an *X* state if the non-zero elements belong to the diagonal and the anti-diagonal [63,64,65]:

(6) $$\rho_{x}=\left(\begin{array}{cccc} \rho_{11} & 0 & 0 & \rho_{14}\\ 0 & \rho_{22} & \rho_{23} & 0\\ 0 & \rho_{32} & \rho_{33} & 0\\ \rho_{41} & 0 & 0 & \rho_{44} \end{array}\right),$$

with $\rho_{jj}$ being real (*j* = 1, 2, 3, 4) and the off-diagonal elements being complex.

On the other hand, we compute the local density operators of the state $\rho^{\left(13\right)}$ which belongs to Alice, and the state $\rho^{\left(24\right)}$, which belongs to Bob, respectively:

(7) $$\begin{array}{ccc} \rho^{\left(13\right)}=\rho^{\left(24\right)} & = & \frac{1}{{\left(1+p^{2}+q^{2}\right)}^{2}}{\left[|\alpha \right|}^{2}\left(1+p^{2}+q^{2}\right)|\;00\;\rangle \langle \;00\;|\;+{\left|\beta \right|}^{2}\left(1+p^{2}+q^{2}\right)\left|\;11\;\rangle \langle \;11\;\right|\\ & & +(p^{2}q^{2}+{\left|\beta \right|}^{2}q^{4}+{\left|\beta \right|}^{2}q^{2}+{\left|\alpha \right|}^{2}p^{4}+{\left|\alpha \right|}^{2}p^{2})|\;01\;\rangle \langle \;01\;|\\ & & +(p^{2}q^{2}+{\left|\beta \right|}^{2}p^{4}+{\left|\beta \right|}^{2}p^{2}+{\left|\alpha \right|}^{2}q^{4}+{\left|\alpha \right|}^{2}q^{2})|\;10\;\rangle \langle \;10\;|\\ & & +\left(pq+p^{3}q+pq^{3}\right)(|\;01\;\rangle \langle \;10\;|+|\;10\;\rangle \langle \;01\;|)]. \end{array}$$

The density operators of the two states are equal, and they are also *X* states.

Two necessary conditions needed to be satisfied in order to say that the input state ${|\psi \rangle }_{12}$ has been broadcast [51] are:

- The local reduced density operators $\rho^{\left(13\right)}$ and $\rho^{\left(24\right)}$ are separable;
- The nonlocal states $\rho^{\left(14\right)}$ and $\rho^{\left(23\right)}$ are inseparable.

**Figure 2:**
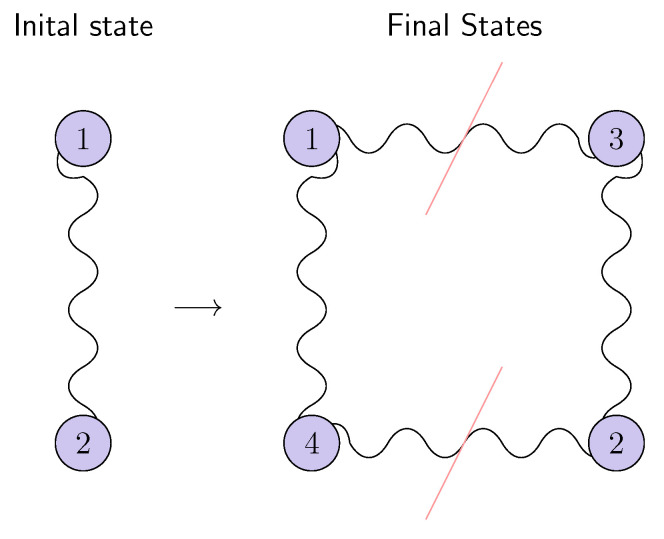
The broadcasting of the entanglement process implies that having an initially pure entangled state ${|\;\psi \;\rangle }^{\left(12\right)}$, we will end up with two entangled mixed states and two separable states.

The broadcasting of entanglement is shown in Figure 2.

We will use the concurrence as a measure of entanglement in this paper [66,67]. The concurrence of two qubits found in an *X* state has the expression [68]:

(8) $$C\left(\rho_{x}\right)=2\;\max\left\{0,|\rho_{23}|-\sqrt{\rho_{11}\;\rho_{44}},\;\left|\rho_{14}\right|-\sqrt{\rho_{22}\;\rho_{33}}\right\}.$$

First, we evaluate the concurrence of the local states $\rho^{\left(13\right)}$ and $\rho^{\left(24\right)}$ and obtain the following, according to (8):

(9) $$C\left(\rho^{\left(13\right)}\right)=C\left(\rho^{\left(24\right)}\right)=2\max\left\{0,\frac{pq-|\alpha |\;|\beta |}{1+p^{2}+q^{2}}\right\}.$$

These local states are separable if |α||β|≥pq, which is equivalent to

(10) $$\frac{1}{2}\left[1-\sqrt{1-4p^{2}{\left(1-p\right)}^{2}}\right]\leq {\left|\alpha \right|}^{2}\leq \frac{1}{2}\left[1+\sqrt{1-4p^{2}{\left(1-p\right)}^{2}}\right].$$

On the other hand, by using Equation (8), we compute the concurrence of the *X* states $\rho^{\left(14\right)}$ and $\rho^{\left(23\right)}$ and found that they are equal:

$$\begin{array}{ccc} & & C\left(\rho^{\left(14\right)}\right)=C\left(\rho^{\left(23\right)}\right)=\;2\;\max\left\{0,\frac{1}{{\left(1+p^{2}+q^{2}\right)}^{2}}[4pq|\alpha ||\beta |-\right.\\ & & \left.-\sqrt{\left({\left|\beta \right|}^{2}p^{4}+{\left|\beta \right|}^{2}p^{2}+{\left|\alpha \right|}^{2}q^{4}+{\left|\alpha \right|}^{2}q^{2}\right)\left({\left|\beta \right|}^{2}q^{4}+{\left|\beta \right|}^{2}q^{2}+{\left|\alpha \right|}^{2}p^{4}+{\left|\alpha \right|}^{2}p^{2}\right)}]\right\}. \end{array}$$

**Figure 3:**
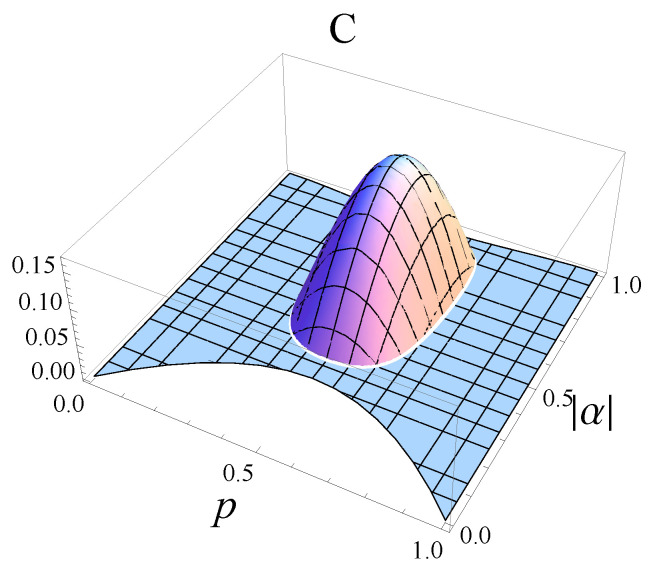
Concurrence of the two output states $\rho^{\left(14\right)}$ and $\rho^{\left(23\right)}$ generated by asymmetric broadcasting of entanglement. We proved that $C\left(\rho^{\left(14\right)}\right)=C\left(\rho^{\left(23\right)}\right)$.

In Figure 3, we plot the concurrence of the output states obtained in the process of asymmetric broadcasting of entanglement in terms of |α|, which characterizes the initial state, and parameter *p*, which describes the asymmetric cloner.

We want now to find the position of the maximum of the concurrence. Therefore, we need to solve the equation $\frac{\partial C}{\partial |\alpha |}=0$. This has the solution $\left|\alpha \right|=\frac{1}{\sqrt{2}}$, i.e., the initial state is a maximally entangled one. In addition, the equation $\frac{\partial C}{\partial p}=0$ leads to the solution $p=\frac{1}{2}$, which corresponds to the case of the symmetric cloning machine.

## 3. The Quantum Discord of the States Obtained by the Local Optimal Universal Asymmetric Cloning Machines

A quite recently introduced measure of quantum correlations is the so-called quantum discord, defined in Ref. [36]. Suppose again that Alice and Bob share two qubits found in the entangled state of Equation (1). Following the protocol of asymmetric broadcasting of entanglement presented in Section 2, they end up with the mixed states $\rho^{\left(14\right)}$ and $\rho^{\left(23\right)}$ given in Equations (4) and (5), respectively. Our purpose here is to analyze the behaviour of the quantum discord of the two output states $\rho^{\left(14\right)}$ and $\rho^{\left(23\right)}$.

In classical information theory, there are two definitions for a concept called mutual information, which are based on the Shannon entropy *H* and the conditional Shannon entropy H(A|B):

(11) $$\begin{array}{ccc} I(A:B) & = & H(A)+H(B)-H(A,B); \end{array}$$

(12) $$\begin{array}{ccc} J(A:B) & = & H(A)-H(A|B). \end{array}$$

An important result in classical information is that the two above definitions (11) and (12) are equivalent. This is not valid in the case of the generalization to the quantum case.

Suppose that two parties share a bipartite quantum system found in the mixed state $\rho_{AB}$. The von Neumann entropy has the expression

$$S\left(\rho \right)=-\mathrm{Tr}\left(\rho \log_{2}\rho \right),$$

and let $\rho_{A(B)}={\mathrm{Tr}}_{B(A)}\;\left(\rho_{AB}\right)$ be the reduced states of the two subsystems. The quantum mutual information between the two subsystems, A and B, was defined in Ref. [36]:

(13) $$I\left(\rho_{AB}\right)=S\left(\rho_{A}\right)+S\left(\rho_{B}\right)-S\left(\rho_{AB}\right).$$

On the other hand, the generalization to the quantum case of the definition (12) is quite difficult, since it involves measurements on the second subsystem *B*. Suppose that these measurements are described by the projectors $\left\{\Pi_{k}^{B}\right\}$. The output state of the subsystem *A*, obtained after the von Neumann measurement on the second subsystem is performed, leading to the result *j*, has the expression [36]:

(14) $$\rho_{A|\Pi_{j}^{B}}=\frac{1}{p_{j}}\;{\mathrm{Tr}}_{B}\left(I⊗\Pi_{j}^{B}\;\rho_{AB}\;I⊗\Pi_{j}^{B}\right),$$

Above $p_{j}$ represents the probability of obtaining the output *j*:

$$p_{j}=\mathrm{Tr}\left(\rho_{AB}\;I⊗\Pi_{j}^{B}\right).$$

The expression of the quantum conditional entropy is given by:

(15) $$S\left(\rho_{A|\{\Pi_{j}^{B}\}}\right)=\sum_{j}p_{j}\;S\left(\rho_{A|\Pi_{j}^{B}}\right).$$

The quantum mutual information, which is the analogue of the classical definition (12), is:

(16) $$J\left(\rho_{AB}\right){|}_{\left\{\Pi_{j}^{B}\right\}}=S\left(\rho_{A}\right)-S\left(\rho_{A|\{\Pi_{j}^{B}\}}\right).$$

A different concept introduced in Refs. [36,37] is the so-called classical correlation, which is obtained by taking the supremum over all the possible measurements on the second subsystem *B*:

(17) $$C_{A}\left(\rho_{AB}\right)=\underset{\left\{\Pi_{j}^{B}\right\}}{\sup}J\left(\rho_{AB}\right){|}_{\left\{\Pi_{j}^{B}\right\}}.$$

Finally, we arrive at the definition of the quantum *A*-discord [36]:

(18) $$D_{A}\left(\rho_{AB}\right)=I\left(\rho_{AB}\right)-C_{A}\left(\rho_{AB}\right).$$

Until now, we have considered only quantum measurements performed on the second subsystem *B*. By performing measurements on the first subsystem, *A*, one leads to the definition of quantum *B*-discord $D_{B}\left(\rho_{AB}\right)$. One can prove that $D_{A}\left(\rho_{AB}\right)\neq D_{B}\left(\rho_{AB}\right)$.

Any *X* state can be brought to its canonical form, where all the elements of the density matrix are real and non-negative, by applying the following local unitary operator [69,70,71,72]:

(19) $$U_{A}⊗U_{B}=e^{-i\;\left(\varphi_{14}+\varphi_{23}\right)\;\sigma_{3}/4}⊗e^{-i\;\left(\varphi_{14}-\varphi_{23}\right)\;\sigma_{3}/4}.$$

Therefore, the canonical form of the density operator $\rho_{x}$ is [69]:

(20) $$\rho_{x}^{can}=U_{A}⊗U_{B}\;\rho_{x}\;U_{A}^{†}⊗U_{B}^{†}=\left(\begin{array}{cccc} \rho_{11} & 0 & 0 & |\rho_{14}|\\ 0 & \rho_{22} & |\rho_{23}| & 0\\ 0 & |\rho_{32}| & \rho_{33} & 0\\ |\rho_{41}| & 0 & 0 & \rho_{44} \end{array}\right).$$

One knows that quantum correlations remain invariant when local unitary operators are applied. In order to evaluate the quantum discord of the output *X* states $\rho^{\left(14\right)}$ and $\rho^{\left(23\right)}$ given in Equations (4) and (5), respectively, and obtained by asymmetric broadcasting of entanglement, a first step is to bring them to their canonical form with the help of the operator (19). Furthermore, we follow the method proposed by Li et al. [62], as one can see in Appendix A.

We compute the five parameters *r*, *s*, $c_{1}$, $c_{2}$, and $c_{3}$ of the two states by using Equation (A2) in Appendix A. First for the state $\rho^{\left(14\right)}$:

$$\begin{array}{ccc} r^{\left(14\right)} & = & \frac{p(2|\alpha {|}^{2}-1)}{p^{2}-p+1}\\ s^{\left(14\right)} & = & \frac{q(2|\alpha {|}^{2}-1)}{p^{2}-p+1}\\ c_{1}^{\left(14\right)} & = & \frac{2pq|\alpha ||\beta |}{{\left(p^{2}-p+1\right)}^{2}}\\ c_{2}^{\left(14\right)} & = & -\frac{2pq|\alpha ||\beta |}{{\left(p^{2}-p+1\right)}^{2}}\\ c_{3}^{\left(14\right)} & = & \frac{pq}{{\left(p^{2}-p+1\right)}^{2}}. \end{array}$$

Second, for the state $\rho^{\left(23\right)}$ we find:

(21) $$\begin{array}{ccc} r^{\left(23\right)} & = & s^{\left(14\right)}\\ s^{\left(23\right)} & = & r^{\left(14\right)}\\ c_{1}^{\left(23\right)} & = & c_{1}^{\left(14\right)}\\ c_{2}^{\left(23\right)} & = & c_{2}^{\left(14\right)}\\ c_{3}^{\left(23\right)} & = & c_{3}^{\left(14\right)}. \end{array}$$

This is equivalent to the replacement r↔s in the expression of $\rho^{\left(14\right)}$, all the other three parameters $c_{1}$, $c_{2}$, and $c_{3}$ remaining invariant:

(22) $$\rho^{\left(23\right)}=\rho^{\left(14\right)}{|}_{r↔s},$$

according to Equation (A1) from the Appendix A.

By using Equation (A3) of the Appendix A, we remark that the eigenvalues of the state $\rho^{\left(14\right)}$ are identical to the eigenvalues of the state $\rho^{\left(23\right)}$. According to the expression (A5) and Equation (21), we arrive at an interesting equivalence:

(23) $$I\left(\rho^{\left(14\right)}\right)=I\left(\rho^{\left(23\right)}\right).$$

The classical correlation $C_{A}\left(\rho_{AB}\right)$ is numerically evaluated, and finally, by employing Equation (18), we obtain the expression of quantum *A*-discord. Due to the fact that the classical correlation is different in the case of the two states: $C_{A}\left(\rho^{\left(14\right)}\right)\neq C_{A}\left(\rho^{\left(23\right)}\right),$ we obtain that the quantum *A*-discord is also distinct for the two states:

$$D_{A}\left(\rho^{\left(14\right)}\right)\neq D_{A}\left(\rho^{\left(23\right)}\right).$$

**Figure 4:**
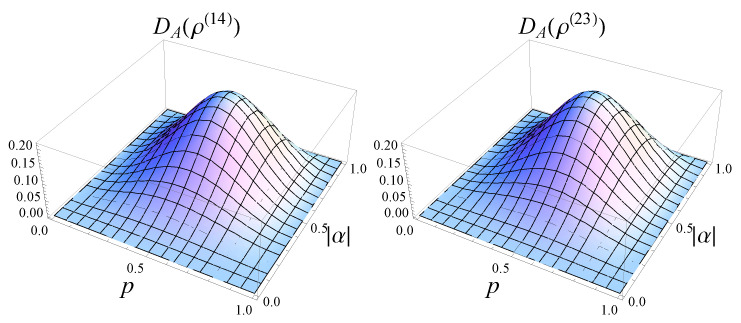
Quantum *A*-discord of the two output states $\rho^{\left(14\right)}$ and $\rho^{\left(23\right)}$ generated by asymmetric broadcasting of entanglement.

The dependence of quantum discord of the states $\rho^{\left(14\right)}$ and $\rho^{\left(23\right)}$ in terms of |α|, which characterizes the initial state, and parameter *p*, which describes the asymmetric cloner, is shown in Figure 4.

The maximum of the quantum *A*-discord was numerically obtained, and it corresponds to $\left|\alpha \right|=\frac{1}{\sqrt{2}}$, i.e., the initial state is a maximally entangled one. On the other hand, the maximum over the parameter *p* is obtained for $p=\frac{1}{2}$, which corresponds to the case of the symmetric cloning machine.

We discussed in Appendix A, that quantum *B*-discord can be obtained from the formula of quantum *A*-discord by performing the replacement r↔s. On the other hand, we have found in Equation (22) that the state $\rho^{\left(23\right)}$ can be determined from the state $\rho^{\left(14\right)}$ also with the replacement r↔s. Therefore, we arrive at the conclusion that

(24) $$\begin{array}{ccc} D_{A}\left(\rho^{\left(14\right)}\right) & = & D_{B}\left(\rho^{\left(23\right)}\right),\\ D_{B}\left(\rho^{\left(14\right)}\right) & = & D_{A}\left(\rho^{\left(23\right)}\right). \end{array}$$

## 4. Consonance

In this section, we propose to investigate a different type of quantum correlation, which was defined in Ref. [73]. Let ρ be the density operator of a bipartite system that is expressed in its general form as:

(25) $$\rho =\sum_{i,j}\sum_{m,n}\rho_{ijmn}\;|\;ij\;\rangle \langle \;mn\;|.$$

The consonance is defined as follows [73]:

(26) $$Cons\left(\rho \right)=\sum_{i,j}\sum_{m,n}|\rho_{ijmn}^{c}\left(1-\delta_{im}\right)\left(1-\delta_{jn}\right),$$

where $\rho^{c}=\left(U_{A}⊗U_{B}\right)\rho \left(U_{A}^{†}⊗U_{B}^{†}\right)$ is obtained such that the local coherence L is canceled. The local coherence was introduced in Ref. [73]:

(27) $$L=\sum_{i\neq m}|\sum_{j=n}\rho_{ijmn}^{c}|+\sum_{j\neq n}|\sum_{i=m}\rho_{ijmn}^{c}|.$$

In the case of *X* mixed states, the local coherence is equal to zero, and therefore, no local unitary operators are applied [73].

We proved that the two output states $\rho^{\left(14\right)}$ and $\rho^{\left(23\right)}$ obtained in the process of broadcasting of entanglement are *X* states. We evaluate the consonance of these states by using definition (26), as well as Equations (4) and (5), and obtain:

(28) $$Cons\left(\rho^{\left(14\right)}\right)=Cons\left(\rho^{\left(23\right)}\right)=\frac{8pq|\alpha |\;|\beta |}{{\left(1+p^{2}+q^{2}\right)}^{2}}.$$

**Figure 5:**
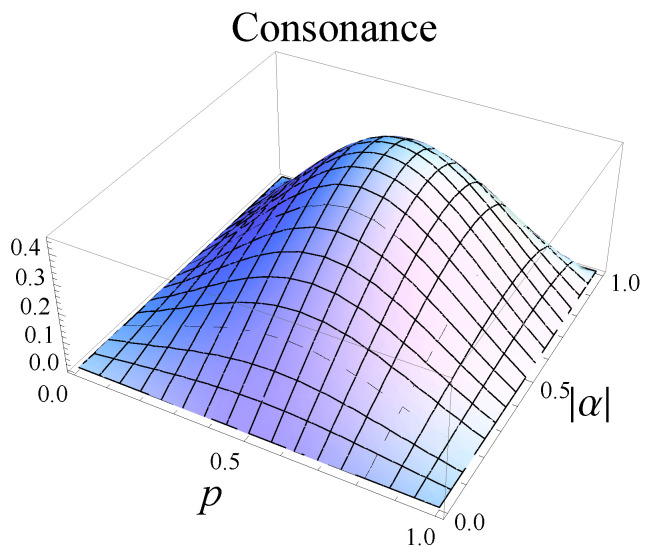
Consonance of the two output states $\rho^{\left(14\right)}$ and $\rho^{\left(23\right)}$ generated by asymmetric broadcasting of entanglement. We proved that $Cons\left(\rho^{\left(14\right)}\right)=Cons\left(\rho^{\left(23\right)}\right)$.

In Figure 5, we plot the consonance of the output states obtained in the process of asymmetric broadcasting of entanglement in terms of the parameters |α| and *p*.

Let us now find the position of the maximum of the consonance. By solving the equation $\frac{\partial Cons}{\partial |\alpha |}=0$, one obtains the solution $\left|\alpha \right|=\frac{1}{\sqrt{2}}$. In addition, the equation $\frac{\partial Cons}{\partial p}=0$ leads to three solutions:

$$p=\frac{1}{2},\;\;p=\frac{1}{2}\left(1-\sqrt{5}\right),\;\;p=\frac{1}{2}\left(1+\sqrt{5}\right).$$

By imposing the physical condition p∈[0,1], we remark that only the solution p=1/2 is valid, which corresponds to the case of symmetric cloning machine.

## 5. A Comparison between the Concurrence, the Quantum A-Discord, and Consonance of the Output States

Our purpose in this section is to make a comparison between the concurrence of the two states obtained in the process of the broadcasting of entanglement (we proved that $C\left(\rho^{\left(14\right)}\right)=C\left(\rho^{\left(23\right)}\right)$), the quantum *A*-discord of the states $\rho^{\left(14\right)}$ and $\rho^{\left(23\right)}$, and consonance (we proved that $Cons\left(\rho^{\left(14\right)}\right)=Cons\left(\rho^{\left(23\right)}\right)$).

**Figure 6:**
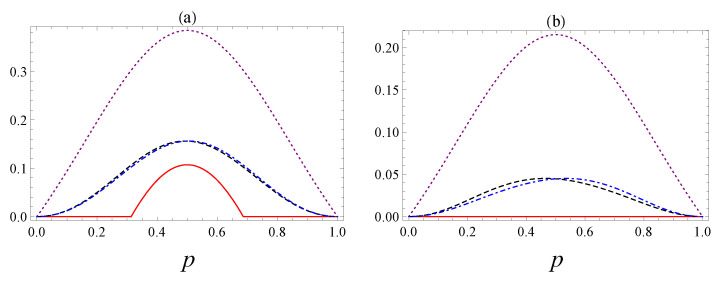
The comparison between concurrence $C\left(\rho^{\left(14\right)}\right)=C\left(\rho^{\left(23\right)}\right)$ (solid red curve), quantum *A*-discord of the state $\rho^{\left(14\right)}$ (black dashed curve), quantum *A*-discord of the state $\rho^{\left(23\right)}$ (blue dot-dashed curve), and consonance $Cons\left(\rho^{\left(14\right)}\right)=Cons\left(\rho^{\left(23\right)}\right)$ (dotted purple curve). We have considered a fixed initial state: (**a**) α=1/2, (**b**) α=1/4.

In Figure 6a, we plot the concurrence, quantum *A*-discord of the state $\rho^{\left(14\right)}$, quantum *A*-discord of the state $\rho^{\left(23\right)}$, and consonance when the parameter is α, which characterizes the initial entangled state is equal to 1/2. We remark that the consonance is larger than the *A*-discord, which is larger than the concurrence. In addition, in the case when α=1/4, we notice in Figure 6b that the states $\rho^{\left(14\right)}$ and $\rho^{\left(23\right)}$ are separable (C=0), and at the same time, their quantum *A*-discord is non-zero, as is their consonance, this being an example of separable states characterized by different kinds of correlation—quantum discord and consonance.

**Figure 7:**
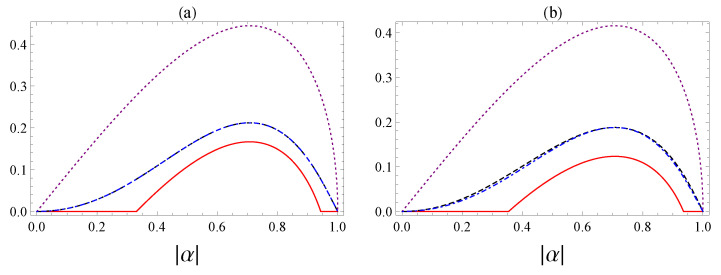
The comparison between concurrence $C\left(\rho^{\left(14\right)}\right)=C\left(\rho^{\left(23\right)}\right)$ (solid red curve), quantum *A*-discord of the state $\rho^{\left(14\right)}$ (black dashed curve), quantum *A*-discord of the state $\rho^{\left(23\right)}$ (blue dot-dashed curve), and consonance $Cons\left(\rho^{\left(14\right)}\right)=Cons\left(\rho^{\left(23\right)}\right)$ (dotted purple curve). We have considered a fixed cloner: (**a**) p=1/2, (**b**) p=0.4.

On the other hand, we present a comparison between concurrence, quantum *A*-discord, and consonance for some fixed values of the parameter *p*, which describes the cloning machine. The case of an optimal universal symmetric cloning machine corresponds to p=1/2, and this is shown in Figure 7a, where we see that the consonance is larger than discord, which is larger than the concurrence. When the symmetric cloning machine is applied, the two output states coincide: $\rho^{\left(14\right)}=\rho^{\left(23\right)}$; therefore, the quantum *A*-discord of the two states is the same. Finally, we plot the concurrence, *A*-discord, and consonance in Figure 7b in the case when the asymmetric cloning machine, characterized by p=0.4, is applied. Again, we have:

(29) $$Cons>D_{A}>C.$$

## 6. Conclusions

In this paper, we have investigated the behaviour of three important types of quantum correlations used in quantum information theory: entanglement, quantum discord, and consonance. The analyzed systems were the two states of the outputs generated in the asymmetric broadcasting of entanglement, denoted by $\rho^{\left(14\right)}$ and $\rho^{\left(23\right)}$.

We have computed the concurrence of the states $\rho^{\left(14\right)}$ and $\rho^{\left(23\right)}$, and we have arrived at the conclusion that:

(30) $$C\left(\rho^{\left(14\right)}\right)=C\left(\rho^{\left(23\right)}\right).$$

Then, we have plotted the concurrence in terms of |α|, which characterizes the initial state and *p*, which denotes the asymmetric cloner.

By using the fact that the state $\rho^{\left(23\right)}$ is obtained from the state $\rho^{\left(14\right)}$ with the replacement r↔s, we have found that:

$$\begin{array}{ccc} D_{A}\left(\rho^{\left(14\right)}\right) & = & D_{B}\left(\rho^{\left(23\right)}\right),\\ D_{B}\left(\rho^{\left(14\right)}\right) & = & D_{A}\left(\rho^{\left(23\right)}\right). \end{array}$$

We have computed the expression of the consonance and plotted it in terms of the parameters |α| and *p*. We have emphasized that the maxima of the three types of correlations—concurrence, discord, and consonance—are obtained when the initial state is a maximally entangled one ($\left|\alpha \right|=1/\sqrt{2}$) and when the symmetric cloning machine is applied (p=1/2).

A detailed comparison of the concurrence, quantum *A*-discord of the state $\rho^{\left(14\right)}$, quantum *A*-discord of the state $\rho^{\left(23\right)}$, and consonance was performed. We have found that the consonance is greater than quantum discord, which is in turn greater than the concurrence.

## Data Availability

Data underlying the results presented in this article may be obtained from the authors upon reasonable request.

## Appendix A. The Evaluation of Quantum Discord for Two Qubits Found in the Canonical Form of the *X* State

In this appendix, we describe the algorithm found by Li et al. [62] for evaluating the quantum discord of two qubits found in the canonical form of the *X* state. The canonical expression of the Fano parameterization of an *X* state is [65,74]:

(A1) $$\rho_{x}^{can}=\frac{1}{4}\;\left(I⊗I+r\;\sigma_{3}⊗I+s\;I⊗\;\sigma_{3}+\sum_{j=1}^{3}c_{j}\;\sigma_{j}⊗\sigma_{j}\right).$$

The parameters *r*, *s*, $c_{1}$, $c_{2}$, and $c_{3}$ are related to the elements of the *X*-state matrix via the formulae [65]:

(A2) $$\begin{array}{ccc} r & = & \rho_{11}+\rho_{22}-\rho_{33}-\rho_{44},\\ s & = & \rho_{11}-\rho_{22}+\rho_{33}-\rho_{44},\\ c_{1} & = & 2\;(|\rho_{23}|+|\rho_{14}|),\\ c_{2} & = & 2\;(|\rho_{23}|-|\rho_{14}|),\\ c_{3} & = & \rho_{11}-\rho_{22}-\rho_{33}+\rho_{44}. \end{array}$$

In Ref. [62], the expression of the eigenvalues of the canonical *X* state (A1) was obtained as follows:

(A3) $$\begin{array}{ccc} \lambda_{1,2} & = & \frac{1}{4}\left[1-c_{3}\pm \sqrt{{\left(r-s\right)}^{2}+{\left(c_{1}+c_{2}\right)}^{2}}\right],\\ \lambda_{3,4} & = & \frac{1}{4}\left[1+c_{3}\pm \sqrt{{\left(r+s\right)}^{2}+{\left(c_{1}-c_{2}\right)}^{2}}\right]. \end{array}$$

For x∈[0,1], we use the following expression for the function:

(A4) $$u\left(x\right)=-\frac{1-x}{2}\;\log_{2}\left(1-x\right)-\frac{1+x}{2}\;\log_{2}\left(1+x\right).$$

The von Neumann entropies associated with the two subsystem have the expressions:

$$\begin{array}{ccc} S(\rho_{A}) & = & 1+u(r);\\ S(\rho_{B}) & = & 1+u(s). \end{array}$$

The quantum mutual information can be computed with the help of Equation (13) and of the above relations:

(A5) $$I\left(\rho_{AB}\right)=2+u\left(r\right)+u\left(s\right)+\sum_{j=1}^{4}\;\lambda_{j}\;\log_{2}\;\lambda_{j}.$$

In Ref. [62], three functions, $f_{1}^{A}$, $f_{2}^{A}$, and $f_{3}^{A}$, were introduced as follows:

$$\begin{array}{ccc} f_{1}^{A} & = & -\frac{1+r+s+c_{3}}{4}\log_{2}\frac{1+r+s+c_{3}}{2(1+s)}-\frac{1-r+s-c_{3}}{4}\log_{2}\frac{1-r+s-c_{3}}{2(1+s)}\\ & & -\frac{1+r-s-c_{3}}{4}\log_{2}\frac{1+r-s-c_{3}}{2(1-s)}-\frac{1-r-s+c_{3}}{4}\log_{2}\frac{1-r-s+c_{3}}{2(1-s)},\\ f_{2}^{A} & = & 1+u\left(\sqrt{r^{2}+c_{1}^{2}}\right),\\ f_{3}^{A} & = & 1+u\left(\sqrt{r^{2}+c_{2}^{2}}\right). \end{array}$$

The classical correlation $C_{A}\left(\rho_{AB}\right)$ can be evaluated with the help of the following formula found by Li et al. [62]:

(A6) $$C_{A}\left(\rho_{AB}\right)=S\left(\rho_{A}\right)-\min\left\{f_{1}^{A},f_{2}^{A},f_{3}^{A}\right\}.$$

In conclusion, after determining the value of the classical correlation, one needs to employ Equation (18) in order to find the quantum *A*-discord of the canonical form of the *X* state.

If we are interested in evaluating the quantum *B*-discord, then we have to make the replacement r↔s in the above approach. We arrive at the conclusion that the quantum *B*-discord is obtained from the expression of *A*-discord $D_{A}$ with the replacement r↔s.
